# Crystal structure and Hirshfeld surface analysis of (*E*)-3-[(4-chloro­benzyl­idene)amino]-5-phenyl­thia­zolidin-2-iminium bromide

**DOI:** 10.1107/S2056989019009885

**Published:** 2019-07-12

**Authors:** Gulnara Sh. Duruskari, Ali N. Khalilov, Mehmet Akkurt, Gunay Z. Mammadova, Taras Chyrka, Abel M. Maharramov

**Affiliations:** a Organic Chemistry Department, Baku State University, Z. Xalilov str. 23, Az, 1148 Baku, Azerbaijan; b Department of Physics and Chemistry, "Composite Materials" Scientific Research Center, Azerbaijan State Economic University (UNEC), H. Aliyev str. 135, Az 1063, Baku, Azerbaijan; c Department of Physics, Faculty of Sciences, Erciyes University, 38039 Kayseri, Turkey; d Department of Theoretical and Industrial Heat Engineering (TPT), National Technical University of Ukraine "Igor Sikorsky Kyiv Polytechnic Institute", 03056, Kyiv, Ukraine

**Keywords:** crystal structure, isotypic, charge-assisted hydrogen bonding, thia­zolidine ring, disorder, Hirshfeld surface analysis

## Abstract

The title salt, C_16_H_15_ClN_3_S^+^·Br^−^, is isotypic with (*E*)-3-[(4-fluoro­benzyl­idene)amino]-5-phenyl­thia­zolidin-2-iminium bromide [Khalilov *et al.* (2019 ▸). *Acta Cryst*. E**75**, 662–666]. In the cation of the title salt, the atoms of the phenyl ring attached to the central thia­zolidine ring and the atom joining the thia­zolidine ring to the benzene ring are disordered over two sets of sites with occupancies of 0.570 (3) and 0.430 (3). The major and minor components of the disordered thia­zolidine ring adopt slightly distorted envelope conformations, with the C atom bearing the phenyl ring as the flap atom. In the crystal, centrosymmetrically related cations and anions are linked into dimeric units *via* N—H⋯Br hydrogen bonds, which are further connected by weak C—H⋯Br contacts into chains parallel to the *a* axis. Furthermore, not existing in the earlier report of (*E*)-3-[(4-fluoro­benzyl­idene)amino]-5-phenyl­thia­zolidin-2-iminium bromide, C—H⋯π inter­actions and π–π stacking inter­actions [centroid-to-centroid distance = 3.897 (2) Å] between the major components of the disordered phenyl ring contribute to the stabilization of the mol­ecular packing. Hirshfeld surface analysis and two-dimensional fingerprint plots indicate that the most important contributions for the crystal packing are from H⋯H (30.5%), Br⋯H/H⋯Br (21.2%), C⋯H/H⋯C (19.2%), Cl⋯H/H⋯Cl (13.0%) and S⋯H/H⋯S (5.0%) inter­actions.

## Abstract 2

In the crystal of the title salt, the cations and anions are linked *via* N—H⋯Br hydrogen bonds. In the ^1^H NMR spectra of this compound, the NH iminium protons are observed at *δ* = 10.46 p.p.m., which confirms the strong charge-assisted hydrogen bonding (CAHB) in the =HN^+^—H**⋯**Br^−^ synthon.

## Chemical context   

The thia­zolidine ring system posses special importance in synthetic and medicinal chemistry. Substituted thia­zolidine derivatives are known to exhibit various biological activities such as anti­viral, anti­cancer, anti-tubercular, and anti­microbial *etc.* (Makwana & Malani 2017 ▸). Schiff bases have been widely used as versatile ligands in the synthesis, catalysis and design of materials (Akbari *et al.*, 2017 ▸; Akkurt *et al.*, 2018 ▸; Asadov *et al.*, 2016 ▸; Gurbanov *et al.*, 2018*a* ▸,*b* ▸; Ma *et al.*, 2017*a* ▸,*b* ▸; Mamedov *et al.*, 2018 ▸). Weak inter­actions, namely hydrogen bonding, π-inter­actions, *etc*. provided by N-containing ligands can also contribute to their structural organization, coordination abilities and catalytic activity, among other properties (Khalilov *et al.*, 2019 ▸; Maharramov *et al.*, 2009 ▸, 2010 ▸; Mahmoudi *et al.*, 2018*a* ▸,*b* ▸; Mahmudov *et al.*, 2014 ▸, 2019 ▸; Mamedov *et al.*, 2015 ▸; Mitoraj *et al.*, 2018 ▸; Shixaliyev *et al.*, 2014 ▸; Zubkov *et al.*, 2018 ▸). As part of our ongoing studies in this field, we report herein the crystal structure and Hirshfeld surface analysis of the title compound, (*E*)-3-[(4-chloro­benzyl­idene)amino]-5-phenyl­thia­zolidin-2-iminium bromide.
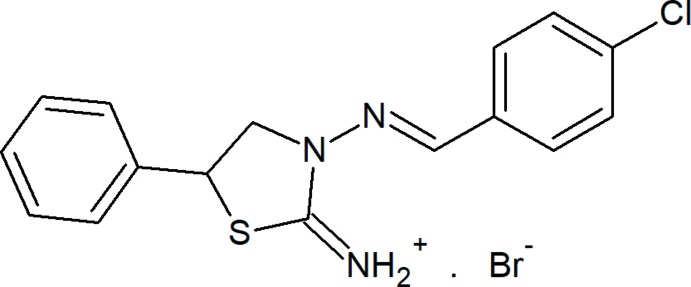


## Structural commentary   

**Figure 1:**
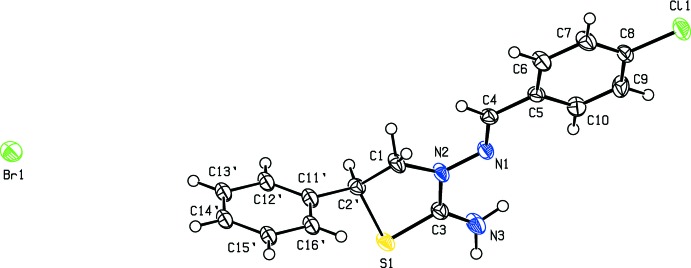
The mol­ecular structure of the title salt. Displacement ellipsoids are drawn at the 50% probability level. H atoms are shown as spheres of arbitrary radius. Only the major component of the disorder is shown for clarity.

The major and minor components (S1/N2/C1/C2′/C3 and S1/N2/C1/C2/C3) of the thia­zolidine ring in the cation of the title salt (Fig. 1 ▸) both adopt a distorted envelope conformation, with puckering parameters *Q*(2) = 0.432 (3) Å, φ(2) = 33.5 (4)° for the major component and *Q*(2) = 0.414 (4) Å, φ(2) = 326.1 (5)° for the minor component. The mean planes of the major and minor components of the disordered thia­zolidine ring make dihedral angles of 14.99 (14), 88.45 (16), 84.3 (2)° and 22.82 (16), 86.85 (18), 83.9 (2)°, respectively, with the chloro­phenyl ring (C5–C10) and the major- and minor-disorder components (C11′–C16′ and C11–C16) of the phenyl ring. The N2—N1—C4—C5 bridge that links the thia­zolidine and 4-chloro­phenyl rings has a torsion angle of 176.4 (2)°.

## Supra­molecular features and Hirshfeld surface analysis   

**Figure 2:**
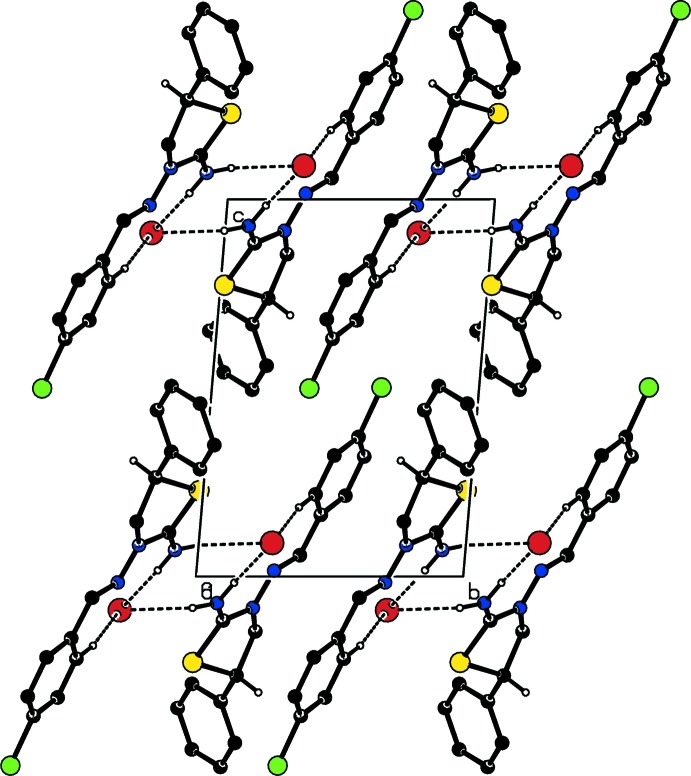
Packing viewed along the *a*-axis direction showing the N—H⋯Br and C—H⋯Br inter­actions (dashed lines).

In the crystal, centrosymmetrically related cations and anions are linked into dimeric units *via* N—H⋯Br hydrogen bonds, which are further connected by weak C—H⋯Br contacts, into chains parallel to the *a*-axis direction (Table 1 ▸; Figs. 2 ▸ and 3 ▸). Furthermore, C—H⋯π inter­actions (Table 1 ▸) and π–π stacking inter­actions [*Cg*4 ⋯*Cg*4(2 − *x*, − *y*, 1 − *z*) = 3.897 (2) Å where *Cg*4 is the centroid of the major component of the disordered phenyl ring] contribute to the stabilization of the mol­ecular packing.

**Figure 4:**
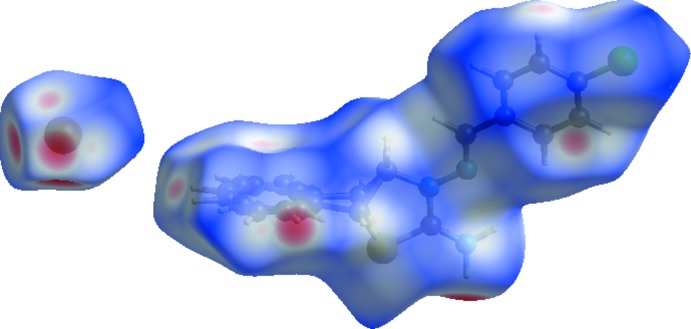
Hirshfeld surface of the title salt mapped with *d_norm_*.

**Figure 5:**
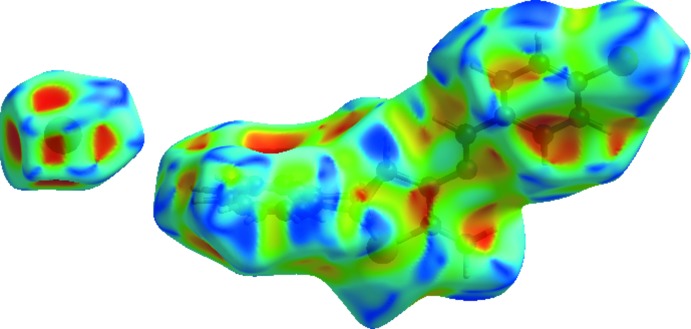
Hirshfeld surface of the title salt mapped with shape-index.

**Figure 6:**
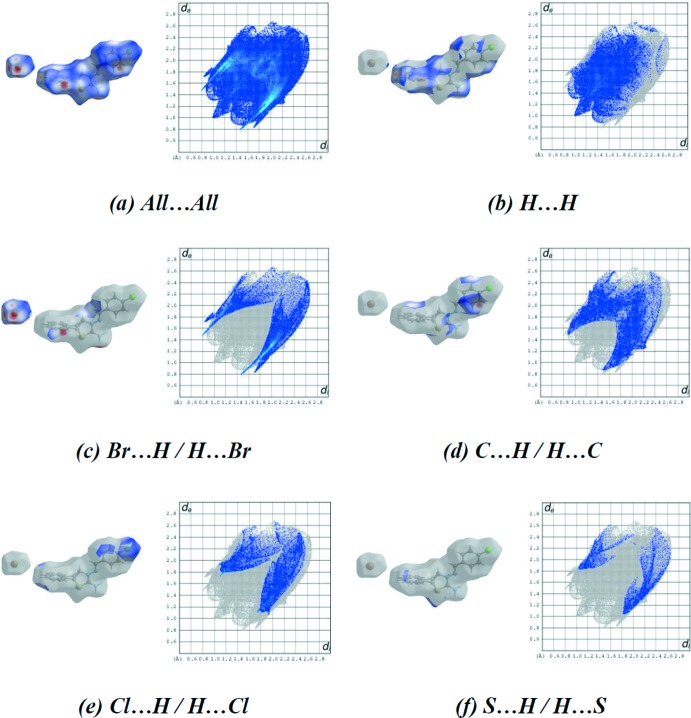
Hirshfeld surface representations and the two-dimensional fingerprint plots of the title salt, showing (*a*) all inter­actions, and delineated into (*b*) H⋯H, (*c*) Br⋯H/H⋯Br, (*d*) C⋯H/H⋯C, (*e*) Cl⋯H/H⋯Cl and (*f*) S⋯H/H⋯S inter­actions [*d*_e_ and *d*_i_ represent the distances from a point on the Hirshfeld surface to the nearest atoms outside (external) and inside (inter­nal) the surface, respectively].

Hirshfeld surface analysis (Spackman & Jayatilaka, 2009 ▸) was used to qu­antify and visualize the inter­molecular inter­actions and to explain the observed crystal packing. *CrystalExplorer3.1* (Wolff *et al.*, 2012 ▸) was used to generate *d*_norm_ surface plots and two-dimensional fingerprint plots (Spackman & McKinnon, 2002 ▸). The Hirshfeld surface mapped over *d*_norm_ using a standard surface resolution with a fixed colour scale of −0.4687 (red) to 1.2270 a.u. (blue) is shown in Fig. 4 ▸. The shape-index of the Hirshfeld surface is a tool to visualize π–π stacking inter­actions by the presence of adjacent red and blue triangles; if there are no adjacent red and/or blue triangles, then there are no π–π inter­actions. Fig. 5 ▸ clearly suggest that there are π–π inter­actions present in the title salt. Fig. 6 ▸ *a* shows the two-dimensional fingerprint for the sum of the contacts contributing to the Hirshfeld surface represented in normal mode (Tables 1 ▸ and 2 ▸). The fingerprint plots delineated into H⋯H (30.5%), Br⋯H/H⋯Br (21.2%), C⋯H/H⋯C (19.2%), Cl⋯H/H⋯Cl (13.0%) and S⋯H/H⋯S (5.0%) inter­actions are shown in Fig. 6 ▸ *b*–*f*, respectively. The most significant inter­molecular inter­actions are the H⋯H inter­actions (30.5%; ig. 6*b*). The various contributions to the Hirshfeld surface are listed in Table 3 ▸.

## Database survey   

A search of the Cambridge Structural Database (CSD, Version 5.40, update of November 2018; Groom *et al.*, 2016 ▸) for 2-thia­zolidiniminium compounds gave eight hits, *viz.* BOBWIB (Khalilov *et al.*, 2019 ▸), UDELUN (Akkurt *et al.*, 2018 ▸), WILBIC (Marthi *et al.*, 1994 ▸), WILBOI (Marthi *et al.*, 1994 ▸), WILBOI01 (Marthi *et al.*, 1994 ▸), YITCEJ (Martem’yanova *et al.*, 1993*a* ▸), YITCAF (Martem’yanova *et al.*, 1993*b* ▸) and YOPLUK (Marthi *et al.*, 1995 ▸).

The structure of BOBWIB (Khalilov *et al.*, 2019 ▸) is isotypic with that of the title salt. In BOBWIB, the phenyl ring is disordered over two sets of sites with a refined occupancy ratio of 0.503 (4):0.497 (4). The mean plane of the thia­zolidine ring makes dihedral angles of 13.51 (14), 48.6 (3) and 76.5 (3)°, respectively, with the fluoro­phenyl ring and the major- and minor-disorder components of the phenyl ring. The central thia­zolidine ring adopts an envelope conformation. In the crystal, centrosymmetrically related cations and anions are linked into dimeric units *via* N—H⋯Br hydrogen bonds, which are further connected by weak C—H⋯Br hydrogen bonds into chains parallel to [110]. In the crystal of UDELUN (Akkurt *et al.*, 2018 ▸), C—H⋯Br and N—H⋯Br hydrogen bonds link the components into a three-dimensional network with the cations and anions stacked along the *b*-axis direction. Weak C—H⋯π inter­actions, which only involve the minor-disorder component of the ring, also contribute to the mol­ecular packing. In addition, there are inversion-related Cl⋯Cl halogen bonds and C—Cl⋯π(ring) contacts. In the remaining structures, the 3-N atom carries a C-atom substituent instead of an N-atom substituent, as found in the title compound. The first three crystal structures were determined for racemic (WILBIC; Marthi *et al.*, 1994 ▸) and two optically active samples (WILBOI and WILBOI01; Marthi *et al.*, 1994 ▸) of 3-(20-chloro-20-phenyl­eth­yl)-2-thia­zolidiniminium *p*-toluene­sulfonate. In all three structures, the most disordered fragment of the mol­ecules is the asymmetric C atom and the Cl atom attached to it. The disorder of the cation in the racemate corresponds to the presence of both enanti­omers at each site in the ratio 0.821 (3):0.179 (3). The system of hydrogen bonds connecting two cations and two anions into 12-membered rings is identical in the racemic and in the optically active crystals. YITCEJ (Martem’yanova *et al.*, 1993*a* ▸) is the product of the inter­action of 2-amino-5-methyl­thia­zoline with methyl iodide, with alkyl­ation at the endocyclic N atom, while YITCAF (Martem’yanova *et al.*, 1993*b* ▸) is the product of the reaction of 3-nitro-5-meth­oxy-, 3-nitro-5- chloro- and 3-bromo-5-nitro­salicyl­aldehyde with the heterocyclic base to form the salt-like complexes.

## Synthesis and crystallization   

To a 1 mmol solution of 3-amino-5-phenyl­thia­zolidin-2-iminium bromide in 20 mL of ethanol was added 1 mmol of 4-chloro­benzaldehyde. The mixture was refluxed for 2 h and then cooled down. The reaction products, precipitated from the reaction mixture as colourless single crystals, were collected by filtration and washed with cold acetone.

**(** ***E*** **)-3-[(4-chloro­benzyl­idene)amino]-5-phenyl­thia­zolidin-2-iminium bromide**: yield 78%, m.p. 531–532 K. Analysis calculated for C_16_H_15_BrClN_3_S (*M*_r_ = 396.73): C 48.44, H 3.81, N 10.59. Found: C 48.40, H 3.78, N 10.55%. ^1^H NMR (300 MHz, DMSO-*d*_6_): 4.56 (*k*, 1H, CH_2_, ^3^ *J*_H–H_ = 6.9); 4.89 (*t*, 1H, CH_2_, ^3^ *J*_H–H_ = 7.8); 5.61 (*t*, 1H, CH—Ar, ^3^ *J*_H–H_ = 7.2); 7.36–8.04 (*m*, 9H, 9Ar—H); 8.47 (*s*, 1H, CH=); 10.46 (*s*, 2H, H_2_N^+^=). ^13^C NMR (75 MHz, DMSO-*d*_6_): 45.40, 55.95, 125.13, 127.77, 128.85, 129.06, 130.49, 131.84, 132.15, 137.40, 149.94, 167.96. MS (ESI), *m*/*z*: 316.82 [C_16_H_15_ClN_3_S]^+^ and 79.88 Br^−^.

## Refinement   

Crystal data, data collection and structure refinement details are summarized in Table 4 ▸. All C-bound H atoms were placed at calculated positions using a riding model, with aromatic C—H = 0.95–1.00 Å, and with *U*_iso_(H) = 1.2*U*_eq_(C). Hydrogen atoms of the amino groups were located directly from difference-Fourier maps and were constrained with AFIX 3 instructions (N—H = 0.90 Å) in order to ensure a chemically reasonable environment for these groups. These hydrogen atoms were modelled with isotropic thermal displacement parameters fixed at 1.2*U*_eq_(N). One outlier (001) was omitted in the final cycles of refinement. The phenyl group and the carbon atom of the 1,3-thia­zolidine group attached to it were refined as positionally disordered over two sets of sites with refined occupancies of 0.570 (3) and 0.430 (3).

## Supplementary Material

Crystal structure: contains datablock(s) I. DOI: 10.1107/S2056989019009885/ff2160sup1.cif

Structure factors: contains datablock(s) I. DOI: 10.1107/S2056989019009885/ff2160Isup2.hkl

Supporting information file. DOI: 10.1107/S2056989019009885/ff2160Isup3.cml

CCDC reference: 1837122

Additional supporting information:  crystallographic information; 3D view; checkCIF report

## Figures and Tables

**Figure 3:**
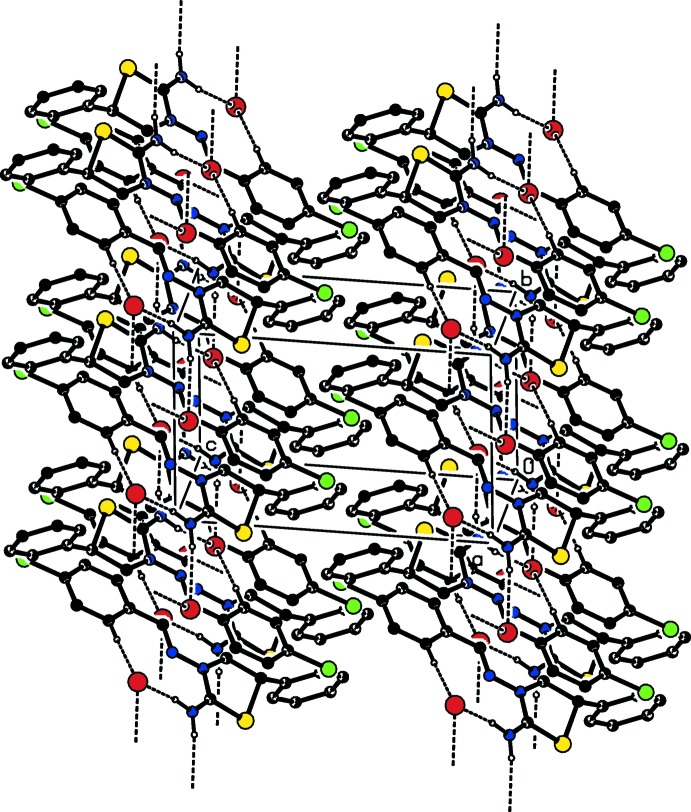
A perspective view of the crystal structure of the title compound.

**Table 1:** Hydrogen-bond geometry (Å, °) *Cg*3 is the centroid of the C5–C10 benzene ring of the chloro­phenyl moiety. *Cg*4 and *Cg*5 are the centroids of the major and minor components of the disordered phenyl ring, respectively.

*D*—H⋯*A*	*D*—H	H⋯*A*	*D*⋯*A*	*D*—H⋯*A*
N3—H3*A*⋯Br1^i^	0.90	2.56	3.390 (2)	154
N3—H3*B*⋯Br1^ii^	0.90	2.38	3.252 (2)	164
C10—H10*A*⋯Br1^i^	0.95	2.91	3.823 (3)	163
C7—H7*A*⋯*Cg*4^iii^	0.95	2.71	3.595 (3)	155
C7—H7*A*⋯*Cg*5^iii^	0.95	2.70	3.568 (3)	153
C13—H13*A*⋯*Cg*3^iv^	0.95	2.97	3.861 (4)	157

Symmetry codes: (i) 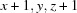; (ii) 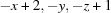; (iii) 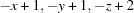; (iv) 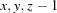.

**Table 2:** Summary of short inter­atomic contacts (Å) in the title salt

Contact	Distance	Symmetry operation
Br1⋯H3*A*	2.56	−1 + *x*, *y*, −1 + *z*
Br1⋯H1*B*	2.56	*x*, *y*, −1 + *z*
Br1⋯H3*B*	2.38	2 − *x*, − *y*, 1 − *z*
Br1⋯H4*A*	2.98	1 − *x*, 1 − *y*, 1 − *z*
Br1⋯H16*A*	2.66	1 − *x*, −*y*, 1 − *z*

**Table 3:** Percentage contributions of inter­atomic contacts to the Hirshfeld surface for the title salt

Contact	Percentage contribution
H⋯H	30.5
Br⋯H/H⋯Br	21.2
C⋯H/H⋯C	19.2
Cl⋯H/H⋯Cl	13.0
S⋯H/H⋯S	5.0
N⋯C/C⋯N	3.3
N⋯H/H⋯N	3.0
C⋯C	2.1
S⋯C/C⋯S	1.7
Br⋯S/S⋯Br	0.4
Cl⋯C/C⋯Cl	0.3
Br⋯C/C⋯Br	0.1
N⋯S/S⋯N	0.1

**Table 4:** Experimental details

Crystal data	
Chemical formula	C_16_H_15_ClN_3_S^+^·Br^−^
*M*_r_	396.73
Crystal system, space group	Triclinic, *P* 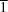
Temperature (K)	150
*a*, *b*, *c* (Å)	8.3146 (5), 8.9424 (5), 12.2388 (6)
α, β, γ (°)	80.988 (2), 76.458 (2), 70.027 (2)
*V* (Å^3^)	828.54 (8)
*Z*	2
Radiation type	Mo *K*α
μ (mm^−1^)	2.77
Crystal size (mm)	0.23 × 0.15 × 0.12

Data collection	
Diffractometer	Bruker APEXII CCD
Absorption correction	Multi-scan (*SADABS*; Bruker, 2003 ▸)
*T*_min_, *T*_max_	0.584, 0.721
No. of measured, independent and observed [*I* > 2σ(*I*)] reflections	13599, 3141, 2768
*R*_int_	0.030
(sin θ/λ)_max_ (Å^−1^)	0.611

Refinement	
*R*[*F* ^2^ > 2σ(*F* ^2^)], *wR*(*F* ^2^), *S*	0.032, 0.075, 1.07
No. of reflections	3141
No. of parameters	167
No. of restraints	13
H-atom treatment	H-atom parameters constrained
Δρ_max_, Δρ_min_ (e Å^−3^)	0.43, −0.32

Computer programs: *APEX2* and *SAINT* (Bruker, 2003 ▸), *SHELXT2014* (Sheldrick, 2015*a* ▸), *SHELXL2016* (Sheldrick, 2015*b* ▸), *ORTEP-3 for Windows* (Farrugia, 2012 ▸) and *PLATON* (Spek, 2003 ▸).

## supplementary crystallographic information

### Crystal data

**Table:**

C_16_H_15_ClN_3_S^+^·Br^−^	*Z* = 2
*M**_r_* = 396.73	*F*(000) = 400
Triclinic, *P*1	*D*_x_ = 1.590 Mg m^−^^3^
*a* = 8.3146 (5) Å	Mo *K*α radiation, λ = 0.71073 Å
*b* = 8.9424 (5) Å	Cell parameters from 5442 reflections
*c* = 12.2388 (6) Å	θ = 2.7–25.6°
α = 80.988 (2)°	µ = 2.77 mm^−^^1^
β = 76.458 (2)°	*T* = 150 K
γ = 70.027 (2)°	Prism, colourless
*V* = 828.54 (8) Å^3^	0.23 × 0.15 × 0.12 mm

### Data collection

**Table:**

Bruker APEXII CCD diffractometer	2768 reflections with *I* > 2σ(*I*)
φ and ω scans	*R*_int_ = 0.030
Absorption correction: multi-scan (SADABS; Bruker, 2003)	θ_max_ = 25.7°, θ_min_ = 2.4°
*T*_min_ = 0.584, *T*_max_ = 0.721	*h* = −10→10
13599 measured reflections	*k* = −10→10
3141 independent reflections	*l* = −14→12

### Refinement

**Table:**

Refinement on *F*^2^	Primary atom site location: structure-invariant direct methods
Least-squares matrix: full	Secondary atom site location: difference Fourier map
*R*[*F*^2^ > 2σ(*F*^2^)] = 0.032	Hydrogen site location: mixed
*wR*(*F*^2^) = 0.075	H-atom parameters constrained
*S* = 1.07	*w* = 1/[σ^2^(*F*_o_^2^) + (0.0318*P*)^2^ + 0.6849*P*] where *P* = (*F*_o_^2^ + 2*F*_c_^2^)/3
3141 reflections	(Δ/σ)_max_ < 0.001
167 parameters	Δρ_max_ = 0.43 e Å^−^^3^
13 restraints	Δρ_min_ = −0.32 e Å^−^^3^

### Special details

**Table:**

Geometry. All esds (except the esd in the dihedral angle between two l.s. planes) are estimated using the full covariance matrix. The cell esds are taken into account individually in the estimation of esds in distances, angles and torsion angles; correlations between esds in cell parameters are only used when they are defined by crystal symmetry. An approximate (isotropic) treatment of cell esds is used for estimating esds involving l.s. planes.

### Fractional atomic coordinates and isotropic or equivalent isotropic displacement parameters (Å^2^)

**Table:**

	*x*	*y*	*z*	*U*_iso_*/*U*_eq_	Occ. (<1)
Br1	0.43584 (3)	0.27419 (3)	0.08917 (3)	0.03616 (11)	
S1	1.16201 (8)	0.01512 (8)	0.77241 (6)	0.02962 (17)	
Cl1	0.71456 (9)	0.63476 (9)	1.50136 (6)	0.03926 (19)	
N1	0.9880 (3)	0.2905 (3)	1.01551 (18)	0.0261 (5)	
N2	1.0069 (3)	0.2228 (3)	0.91692 (18)	0.0295 (5)	
N3	1.2932 (3)	0.0869 (3)	0.9296 (2)	0.0400 (6)	
H3A	1.292718	0.144940	0.983551	0.048*	
H3B	1.384318	−0.002770	0.916811	0.048*	
C1	0.8687 (3)	0.2393 (3)	0.8545 (2)	0.0341 (7)	
H1A	0.816300	0.353542	0.839939	0.041*	
H1B	0.760230	0.250582	0.908539	0.041*	
C2	0.9229 (7)	0.0699 (7)	0.8065 (4)	0.0235 (7)	0.430 (3)
H2A	0.885845	−0.008237	0.866227	0.028*	0.430 (3)
C2'	0.9748 (5)	0.1827 (5)	0.7375 (3)	0.0235 (7)	0.570 (3)
H2'A	1.017275	0.270076	0.692759	0.028*	0.570 (3)
C3	1.1593 (3)	0.1148 (3)	0.8835 (2)	0.0254 (6)	
C4	0.8369 (3)	0.3820 (3)	1.0563 (2)	0.0241 (5)	
H4A	0.743146	0.407061	1.017588	0.029*	
C5	0.8106 (3)	0.4475 (3)	1.1638 (2)	0.0223 (5)	
C6	0.6438 (3)	0.5335 (3)	1.2143 (2)	0.0325 (6)	
H6A	0.549467	0.552836	1.177051	0.039*	
C7	0.6126 (3)	0.5916 (3)	1.3182 (2)	0.0326 (6)	
H7A	0.497686	0.649414	1.353033	0.039*	
C8	0.7509 (3)	0.5642 (3)	1.3698 (2)	0.0263 (6)	
C9	0.9184 (3)	0.4796 (3)	1.3208 (2)	0.0328 (6)	
H9A	1.012263	0.461561	1.358178	0.039*	
C10	0.9491 (3)	0.4214 (3)	1.2178 (2)	0.0291 (6)	
H10A	1.064338	0.363690	1.183537	0.035*	
C11	0.8465 (6)	0.0806 (7)	0.7014 (3)	0.0262 (4)	0.430 (3)
C12	0.8842 (5)	0.1707 (5)	0.6013 (5)	0.0262 (4)	0.430 (3)
H12A	0.959618	0.232164	0.595388	0.031*	0.430 (3)
C13	0.8115 (6)	0.1709 (5)	0.5099 (3)	0.0262 (4)	0.430 (3)
H13A	0.837260	0.232457	0.441512	0.031*	0.430 (3)
C14	0.7011 (6)	0.0810 (6)	0.5186 (3)	0.0262 (4)	0.430 (3)
H14A	0.651415	0.081077	0.456168	0.031*	0.430 (3)
C15	0.6634 (6)	−0.0091 (5)	0.6188 (4)	0.0262 (4)	0.430 (3)
H15A	0.587926	−0.070596	0.624700	0.031*	0.430 (3)
C16	0.7361 (6)	−0.0093 (5)	0.7102 (3)	0.0262 (4)	0.430 (3)
H16A	0.710282	−0.070891	0.778578	0.031*	0.430 (3)
C11'	0.8723 (4)	0.1315 (5)	0.6690 (3)	0.0262 (4)	0.570 (3)
C12'	0.8655 (4)	0.1978 (4)	0.5589 (3)	0.0262 (4)	0.570 (3)
H12B	0.922655	0.274751	0.527276	0.031*	0.570 (3)
C13'	0.7752 (5)	0.1515 (4)	0.49515 (18)	0.0262 (4)	0.570 (3)
H13B	0.770646	0.196839	0.419913	0.031*	0.570 (3)
C14'	0.6917 (4)	0.0390 (4)	0.5415 (3)	0.0262 (4)	0.570 (3)
H14B	0.629962	0.007328	0.497865	0.031*	0.570 (3)
C15'	0.6984 (5)	−0.0273 (3)	0.6515 (3)	0.0262 (4)	0.570 (3)
H15B	0.641287	−0.104273	0.683180	0.031*	0.570 (3)
C16'	0.7887 (5)	0.0189 (4)	0.71531 (18)	0.0262 (4)	0.570 (3)
H16B	0.793296	−0.026363	0.790545	0.031*	0.570 (3)

### Atomic displacement parameters (Å^2^)

**Table:**

	*U*^11^	*U*^22^	*U*^33^	*U*^12^	*U*^13^	*U*^23^
Br1	0.02235 (16)	0.03754 (17)	0.0473 (2)	−0.00105 (11)	−0.01050 (12)	−0.01367 (13)
S1	0.0215 (3)	0.0316 (4)	0.0341 (4)	0.0013 (3)	−0.0070 (3)	−0.0180 (3)
Cl1	0.0365 (4)	0.0458 (4)	0.0323 (4)	0.0007 (3)	−0.0103 (3)	−0.0202 (3)
N1	0.0235 (11)	0.0293 (12)	0.0248 (11)	−0.0027 (9)	−0.0063 (9)	−0.0111 (9)
N2	0.0204 (11)	0.0349 (13)	0.0309 (12)	0.0048 (9)	−0.0100 (9)	−0.0196 (10)
N3	0.0238 (12)	0.0452 (15)	0.0480 (15)	0.0093 (11)	−0.0152 (11)	−0.0292 (12)
C1	0.0207 (13)	0.0397 (16)	0.0382 (16)	0.0095 (12)	−0.0123 (12)	−0.0264 (13)
C2	0.0187 (17)	0.0239 (18)	0.0273 (19)	−0.0033 (14)	−0.0044 (15)	−0.0087 (14)
C2'	0.0187 (17)	0.0239 (18)	0.0273 (19)	−0.0033 (14)	−0.0044 (15)	−0.0087 (14)
C3	0.0220 (13)	0.0265 (13)	0.0274 (14)	−0.0039 (11)	−0.0055 (11)	−0.0089 (11)
C4	0.0190 (13)	0.0218 (13)	0.0307 (14)	−0.0007 (10)	−0.0079 (11)	−0.0083 (11)
C5	0.0201 (12)	0.0201 (12)	0.0260 (13)	−0.0035 (10)	−0.0052 (10)	−0.0057 (10)
C6	0.0201 (13)	0.0372 (16)	0.0405 (16)	0.0008 (11)	−0.0106 (12)	−0.0200 (13)
C7	0.0202 (13)	0.0358 (15)	0.0401 (16)	0.0000 (11)	−0.0053 (12)	−0.0190 (13)
C8	0.0273 (14)	0.0260 (13)	0.0244 (13)	−0.0033 (11)	−0.0051 (11)	−0.0103 (11)
C9	0.0225 (14)	0.0439 (17)	0.0311 (15)	−0.0019 (12)	−0.0125 (12)	−0.0097 (13)
C10	0.0185 (13)	0.0348 (15)	0.0288 (14)	0.0001 (11)	−0.0031 (11)	−0.0100 (12)
C11	0.0219 (8)	0.0310 (9)	0.0269 (9)	−0.0047 (6)	−0.0062 (6)	−0.0127 (7)
C12	0.0219 (8)	0.0310 (9)	0.0269 (9)	−0.0047 (6)	−0.0062 (6)	−0.0127 (7)
C13	0.0219 (8)	0.0310 (9)	0.0269 (9)	−0.0047 (6)	−0.0062 (6)	−0.0127 (7)
C14	0.0219 (8)	0.0310 (9)	0.0269 (9)	−0.0047 (6)	−0.0062 (6)	−0.0127 (7)
C15	0.0219 (8)	0.0310 (9)	0.0269 (9)	−0.0047 (6)	−0.0062 (6)	−0.0127 (7)
C16	0.0219 (8)	0.0310 (9)	0.0269 (9)	−0.0047 (6)	−0.0062 (6)	−0.0127 (7)
C11'	0.0219 (8)	0.0310 (9)	0.0269 (9)	−0.0047 (6)	−0.0062 (6)	−0.0127 (7)
C12'	0.0219 (8)	0.0310 (9)	0.0269 (9)	−0.0047 (6)	−0.0062 (6)	−0.0127 (7)
C13'	0.0219 (8)	0.0310 (9)	0.0269 (9)	−0.0047 (6)	−0.0062 (6)	−0.0127 (7)
C14'	0.0219 (8)	0.0310 (9)	0.0269 (9)	−0.0047 (6)	−0.0062 (6)	−0.0127 (7)
C15'	0.0219 (8)	0.0310 (9)	0.0269 (9)	−0.0047 (6)	−0.0062 (6)	−0.0127 (7)
C16'	0.0219 (8)	0.0310 (9)	0.0269 (9)	−0.0047 (6)	−0.0062 (6)	−0.0127 (7)

### Geometric parameters (Å, º)

**Table:**

S1—C3	1.731 (3)	C8—C9	1.379 (4)
S1—C2'	1.835 (4)	C9—C10	1.374 (4)
S1—C2	1.838 (5)	C9—H9A	0.9500
Cl1—C8	1.744 (3)	C10—H10A	0.9500
N1—C4	1.276 (3)	C11—C12	1.3900
N1—N2	1.386 (3)	C11—C16	1.3900
N2—C3	1.323 (3)	C12—C13	1.3900
N2—C1	1.478 (3)	C12—H12A	0.9500
N3—C3	1.296 (3)	C13—C14	1.3900
N3—H3A	0.8999	C13—H13A	0.9500
N3—H3B	0.9000	C14—C15	1.3900
C1—C2'	1.554 (5)	C14—H14A	0.9500
C1—C2	1.591 (6)	C15—C16	1.3900
C1—H1A	0.9700	C15—H15A	0.9500
C1—H1B	0.9700	C16—H16A	0.9500
C2—C11	1.5386 (19)	C11'—C12'	1.3900
C2—H2A	1.0000	C11'—C16'	1.3900
C2'—C11'	1.5363 (19)	C12'—C13'	1.3900
C2'—H2'A	1.0000	C12'—H12B	0.9500
C4—C5	1.463 (3)	C13'—C14'	1.3900
C4—H4A	0.9500	C13'—H13B	0.9500
C5—C6	1.383 (4)	C14'—C15'	1.3900
C5—C10	1.394 (4)	C14'—H14B	0.9500
C6—C7	1.384 (4)	C15'—C16'	1.3900
C6—H6A	0.9500	C15'—H15B	0.9500
C7—C8	1.371 (4)	C16'—H16B	0.9500
C7—H7A	0.9500		
			
C3—S1—C2'	88.72 (12)	C7—C8—C9	121.5 (2)
C3—S1—C2	90.61 (15)	C7—C8—Cl1	119.5 (2)
C4—N1—N2	117.6 (2)	C9—C8—Cl1	119.0 (2)
C3—N2—N1	116.3 (2)	C10—C9—C8	119.8 (2)
C3—N2—C1	115.9 (2)	C10—C9—H9A	120.1
N1—N2—C1	127.2 (2)	C8—C9—H9A	120.1
C3—N3—H3A	122.6	C9—C10—C5	119.8 (2)
C3—N3—H3B	119.8	C9—C10—H10A	120.1
H3A—N3—H3B	116.8	C5—C10—H10A	120.1
N2—C1—C2'	102.7 (2)	C12—C11—C16	120.0
N2—C1—C2	104.0 (2)	C12—C11—C2	124.0 (5)
N2—C1—H1A	104.7	C16—C11—C2	116.0 (5)
C2'—C1—H1A	104.2	C13—C12—C11	120.0
C2—C1—H1A	145.2	C13—C12—H12A	120.0
N2—C1—H1B	108.2	C11—C12—H12A	120.0
C2'—C1—H1B	145.6	C12—C13—C14	120.0
C2—C1—H1B	106.5	C12—C13—H13A	120.0
H1A—C1—H1B	82.5	C14—C13—H13A	120.0
C11—C2—C1	112.0 (4)	C15—C14—C13	120.0
C11—C2—S1	111.7 (3)	C15—C14—H14A	120.0
C1—C2—S1	101.9 (3)	C13—C14—H14A	120.0
C11—C2—H2A	110.3	C16—C15—C14	120.0
C1—C2—H2A	110.3	C16—C15—H15A	120.0
S1—C2—H2A	110.3	C14—C15—H15A	120.0
C11'—C2'—C1	114.0 (3)	C15—C16—C11	120.0
C11'—C2'—S1	111.5 (3)	C15—C16—H16A	120.0
C1—C2'—S1	103.5 (2)	C11—C16—H16A	120.0
C11'—C2'—H2'A	109.2	C12'—C11'—C16'	120.0
C1—C2'—H2'A	109.2	C12'—C11'—C2'	119.2 (3)
S1—C2'—H2'A	109.2	C16'—C11'—C2'	120.8 (3)
N3—C3—N2	123.1 (2)	C11'—C12'—C13'	120.0
N3—C3—S1	123.5 (2)	C11'—C12'—H12B	120.0
N2—C3—S1	113.40 (18)	C13'—C12'—H12B	120.0
N1—C4—C5	118.8 (2)	C14'—C13'—C12'	120.0
N1—C4—H4A	120.6	C14'—C13'—H13B	120.0
C5—C4—H4A	120.6	C12'—C13'—H13B	120.0
C6—C5—C10	119.4 (2)	C15'—C14'—C13'	120.0
C6—C5—C4	119.2 (2)	C15'—C14'—H14B	120.0
C10—C5—C4	121.4 (2)	C13'—C14'—H14B	120.0
C5—C6—C7	120.9 (2)	C14'—C15'—C16'	120.0
C5—C6—H6A	119.5	C14'—C15'—H15B	120.0
C7—C6—H6A	119.5	C16'—C15'—H15B	120.0
C8—C7—C6	118.6 (2)	C15'—C16'—C11'	120.0
C8—C7—H7A	120.7	C15'—C16'—H16B	120.0
C6—C7—H7A	120.7	C11'—C16'—H16B	120.0
			
C4—N1—N2—C3	−172.7 (2)	C7—C8—C9—C10	0.2 (4)
C4—N1—N2—C1	−2.6 (4)	Cl1—C8—C9—C10	179.2 (2)
C3—N2—C1—C2'	−26.2 (3)	C8—C9—C10—C5	−0.4 (4)
N1—N2—C1—C2'	163.7 (3)	C6—C5—C10—C9	0.8 (4)
C3—N2—C1—C2	25.4 (4)	C4—C5—C10—C9	−177.5 (3)
N1—N2—C1—C2	−144.8 (3)	C1—C2—C11—C12	61.3 (5)
N2—C1—C2—C11	−155.9 (4)	S1—C2—C11—C12	−52.3 (5)
N2—C1—C2—S1	−36.4 (3)	C1—C2—C11—C16	−119.8 (4)
C3—S1—C2—C11	152.1 (4)	S1—C2—C11—C16	126.5 (3)
C3—S1—C2—C1	32.4 (2)	C16—C11—C12—C13	0.0
N2—C1—C2'—C11'	159.8 (3)	C2—C11—C12—C13	178.8 (5)
N2—C1—C2'—S1	38.5 (3)	C11—C12—C13—C14	0.0
C3—S1—C2'—C11'	−157.6 (3)	C12—C13—C14—C15	0.0
C3—S1—C2'—C1	−34.6 (2)	C13—C14—C15—C16	0.0
N1—N2—C3—N3	−9.0 (4)	C14—C15—C16—C11	0.0
C1—N2—C3—N3	179.8 (3)	C12—C11—C16—C15	0.0
N1—N2—C3—S1	171.00 (18)	C2—C11—C16—C15	−178.9 (4)
C1—N2—C3—S1	−0.2 (3)	C1—C2'—C11'—C12'	127.7 (3)
C2'—S1—C3—N3	−158.3 (3)	S1—C2'—C11'—C12'	−115.5 (3)
C2—S1—C3—N3	159.2 (3)	C1—C2'—C11'—C16'	−53.1 (4)
C2'—S1—C3—N2	21.7 (2)	S1—C2'—C11'—C16'	63.7 (3)
C2—S1—C3—N2	−20.8 (3)	C16'—C11'—C12'—C13'	0.0
N2—N1—C4—C5	176.4 (2)	C2'—C11'—C12'—C13'	179.2 (3)
N1—C4—C5—C6	−173.6 (3)	C11'—C12'—C13'—C14'	0.0
N1—C4—C5—C10	4.7 (4)	C12'—C13'—C14'—C15'	0.0
C10—C5—C6—C7	−1.0 (4)	C13'—C14'—C15'—C16'	0.0
C4—C5—C6—C7	177.3 (3)	C14'—C15'—C16'—C11'	0.0
C5—C6—C7—C8	0.8 (4)	C12'—C11'—C16'—C15'	0.0
C6—C7—C8—C9	−0.5 (4)	C2'—C11'—C16'—C15'	−179.2 (3)
C6—C7—C8—Cl1	−179.4 (2)		

### Hydrogen-bond geometry (Å, º)

*Cg*3 is the centroid of the C5–C10 benzene ring of the chlorophenyl moiety. *Cg*4 and *Cg*5 are the centroids of the major and minor components of the disordered phenyl ring, respectively.

**Table:**

*D*—H···*A*	*D*—H	H···*A*	*D*···*A*	*D*—H···*A*
N3—H3*A*···Br1^i^	0.90	2.56	3.390 (2)	154
N3—H3*B*···Br1^ii^	0.90	2.38	3.252 (2)	164
C10—H10*A*···Br1^i^	0.95	2.91	3.823 (3)	163
C7—H7*A*···*Cg*4^iii^	0.95	2.71	3.595 (3)	155
C7—H7*A*···*Cg*5^iii^	0.95	2.70	3.568 (3)	153
C13—H13*A*···*Cg*3^iv^	0.95	2.97	3.861 (4)	157

Symmetry codes: (i) *x*+1, *y*, *z*+1; (ii) −*x*+2, −*y*, −*z*+1; (iii) −*x*+1, −*y*+1, −*z*+2; (iv) *x*, *y*, *z*−1.
